# SimPET—An open online platform for the Monte Carlo simulation of realistic brain PET data. Validation for ^18^F‐FDG scans

**DOI:** 10.1002/mp.14838

**Published:** 2021-03-30

**Authors:** José Paredes‐Pacheco, Francisco Javier López‐González, Jesús Silva‐Rodríguez, Nikos Efthimiou, Aida Niñerola‐Baizán, Álvaro Ruibal, Núria Roé‐Vellvé, Pablo Aguiar

**Affiliations:** ^1^ Radiology and Psychiatry Department Faculty of Medicine Universidade de Santiago de Compostela Galicia Spain; ^2^ Molecular Imaging Unit Centro de Investigaciones Médico‐Sanitarias General Foundation of the University of Málaga Málaga Spain; ^3^ Nuclear Medicine Department & Molecular Imaging Research Group University Hospital (SERGAS) & Health Research Institute of Santiago de Compostela (IDIS) Galicia Spain; ^4^ R&D Department Qubiotech Health Intelligence SL, A Coruña Galicia Spain; ^5^ Positron Emission Tomography Research Centre University of Hull Hull HU6 7RX UK; ^6^ Nuclear Medicine Department Hospital Clinic Barcelona Universitat de Barcelona Barcelona Spain; ^7^ Biomedical Research Networking Center of Bioengineering Biomaterials and Nanomedicine (CIBER‐BBN) Barcelona Spain

**Keywords:** Monte Carlo, PET, quantification, simulation, standardization

## Abstract

**Purpose:**

SimPET (www.sim‐pet.org) is a free cloud‐based platform for the generation of realistic brain positron emission tomography (PET) data. In this work, we introduce the key features of the platform. In addition, we validate the platform by performing a comparison between simulated healthy brain FDG‐PET images and real healthy subject data for three commercial scanners (GE Advance NXi, GE Discovery ST, and Siemens Biograph mCT).

**Methods:**

The platform provides a graphical user interface to a set of automatic scripts taking care of the code execution for the phantom generation, simulation (SimSET), and tomographic image reconstruction (STIR). We characterize the performance using activity and attenuation maps derived from PET/CT and MRI data of 25 healthy subjects acquired with a GE Discovery ST. We then use the created maps to generate synthetic data for the GE Discovery ST, the GE Advance NXi, and the Siemens Biograph mCT. The validation was carried out by evaluating Bland‐Altman differences between real and simulated images for each scanner. In addition, SPM voxel‐wise comparison was performed to highlight regional differences. Examples for amyloid PET and for the generation of ground‐truth pathological patients are included.

**Results:**

The platform can be efficiently used for generating realistic simulated FDG‐PET images in a reasonable amount of time. The validation showed small differences between SimPET and acquired FDG‐PET images, with errors below 10% for 98.09% (GE Discovery ST), 95.09% (GE Advance NXi), and 91.35% (Siemens Biograph mCT) of the voxels. Nevertheless, our SPM analysis showed significant regional differences between the simulated images and real healthy patients, and thus, the use of the platform for converting control subject databases between different scanners requires further investigation.

**Conclusions:**

The presented platform can potentially allow scientists in clinical and research settings to perform MC simulation experiments without the need for high‐end hardware or advanced computing knowledge and in a reasonable amount of time.

## 1 INTRODUCTION

Positron emission tomography (PET) has been widely used in neurology to study brain metabolism, receptor binding, and alterations in regional blood flow.^1^ In particular, ^18^F‐fluorodeoxyglucose (^18^F‐FDG) PET provides images of the global and regional brain glucose consumption, which are of great interest in the clinical diagnosis and follow‐up of neurological disorders such as epilepsy and different forms of dementia.^2^

Despite the fact that brain FDG‐PET images are typically interpreted through visual inspection and manual annotation,^3^ several studies have highlighted the potential benefits of semiquantitative approaches for improving the diagnostic confidence and accuracy in dementia,^4^ epilepsy,^5^ or atypical parkinsonian syndromes.^6^ This has led to the gradual introduction of commercial software for semiquantitative analysis into the clinical routine.^7^ However, the generalized use of quantification has been hampered by the lack of the standardization between quantification methods.^8^, ^9^ The chief obstacle for such standardization has been the lack of reliable and easy‐to‐use ground‐truth references. The most common approach is the use of geometric^10^ or anthropomorphic phantoms,^11^ of which the most popular is the Hoffman phantom.^12^, ^13^, ^14^ However, the use of physical phantoms provides little flexibility for changing shapes and volumes of the brain regions, leading to unrealistic images. An alternative is the use of Monte Carlo (MC) or analytical simulations. Several toolkits exist for MC simulation, such as the Geant4 Application for Tomographic Emission (GATE),^15^ Simulation System for Emission Tomography (SimSET),^16^ or PeneloPET.^17^ The simulated data can then be reconstructed to generate PET images, which can be used to validate the quantification methods using the original digital phantoms as ground truth.^18^, ^19^, ^20^, ^21^, ^22^, ^23^, ^24^ For this, different brain digital phantoms such as the Zubal,^25^ the XCAT brain,^26^ the BigBrain atlas,^27^ and the digital Hoffman^28^ are available, but these have, in general, similar limitations in terms of changing shapes and volumes similar to those of the physical phantoms. These limitations can be overcome by deriving the synthetic phantoms from patient data,^29^, ^30^ allowing the generation of large numbers of different phantoms incorporating voxel‐wise physiological variability. However, the use of all the aforementioned requires a solid background in particle physics, statistics and/or programming as well as access to high computing power, making MC simulation often inaccessible to researchers outside the specialized community which implements these software tools, thus limiting its outreach to clinical facilities, where quantitative methodologies must be validated.

In this work, we present SimPET (www.sim‐pet.org), a free, easy‐to‐use, cloud‐based platform for the generation of synthetic PET images with a special focus on brain imaging. The platform allows the automatic generation of realistic digital brain phantoms derived from patient PET/CT and MRI images by using the Brain‐VISET (voxel‐based iterative simulation for emission tomography) method previously published by our group,^29^ and the simulation and reconstruction of these or other user‐defined phantoms using several validated scanner models included in the platform.

## 2 MATERIALS AND METHODS

### 2.A The SimPET platform

#### 2.A.1 Architecture and deployment

**Fig. 1:**
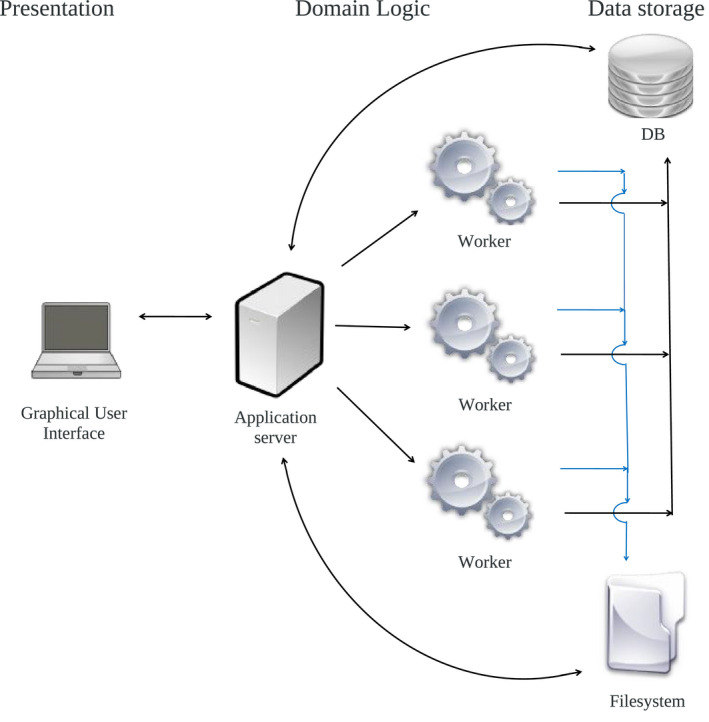
System architecture of SimPET with the three layers: Presentation, domain logic, and data storage.

The SimPET platform is an adaptation from Neurocloud®, a commercial online platform hosting quantification tools (https://qubiotech.com/en/neurocloud) (Qubiotech Health Intelligence SL, A Coruña, Spain). It has a multilayered architecture, with three main layers: presentation (GUI), domain logic, and data storage (Fig. 1). Each layer is built with its own technologies and can be independently upgraded, debugged, and repaired.

The web portal (www.sim‐pet.org) provides a simple graphical user interface (GUI). The application manages the web server, computational processes, and file storage. The platform distributes load to parallel processing “workers" that perform the simulation/reconstruction. The underlying scripts are written in various programming languages, such as Bash, MATLAB, C, and Python. The source code is open, free, and can be downloaded from GitHub (https://github.com/txusser/brainviset_simset). Various well‐validated libraries are used under‐the‐hood, such as SimSET (https://depts.washington.edu/simset), STIR (http://stir.sourceforge.net/),^31^ the Statistical Parametric Mapping package (SPM12),^32^ FSL,^33^ and the Python NiBabel.^34^ The interested reader can find past use cases in our published work.^20^, ^21^, ^35^, ^36^, ^37^

The whole system (including the web application + two simulation workers) is currently deployed on a Lenovo ThinkStation P920 with two Intel Zeon Silver 4114 processors (twenty 2.20 GHz cores and forty threads in total), 126 GB RAM and 6 TB disk space in a mirroring configuration using Ubuntu 16.04 LTS. In addition, thanks to the platform features inherited from Neurocloud, the platform is ready to be deployed in a full cloud‐computing configuration, with the ability of managing autoscaling of the number of simulation cores depending on the load.

#### 2.A.2 Input and output

The platform accepts inputs in the Digital Imaging and Communications in Medicine (DICOM) and NifTI‐1 formats. When uploading DICOM, the images are anonymized and converted to NifTI‐1, which is a more convenient format for mathematical manipulation. The platform outputs the generated Brain‐VISET digital brain phantoms (activity and attenuation maps), sinograms, and reconstructed PET images in NifTI‐1.

#### 2.A.3 Graphical user interface

**Fig. 2:**
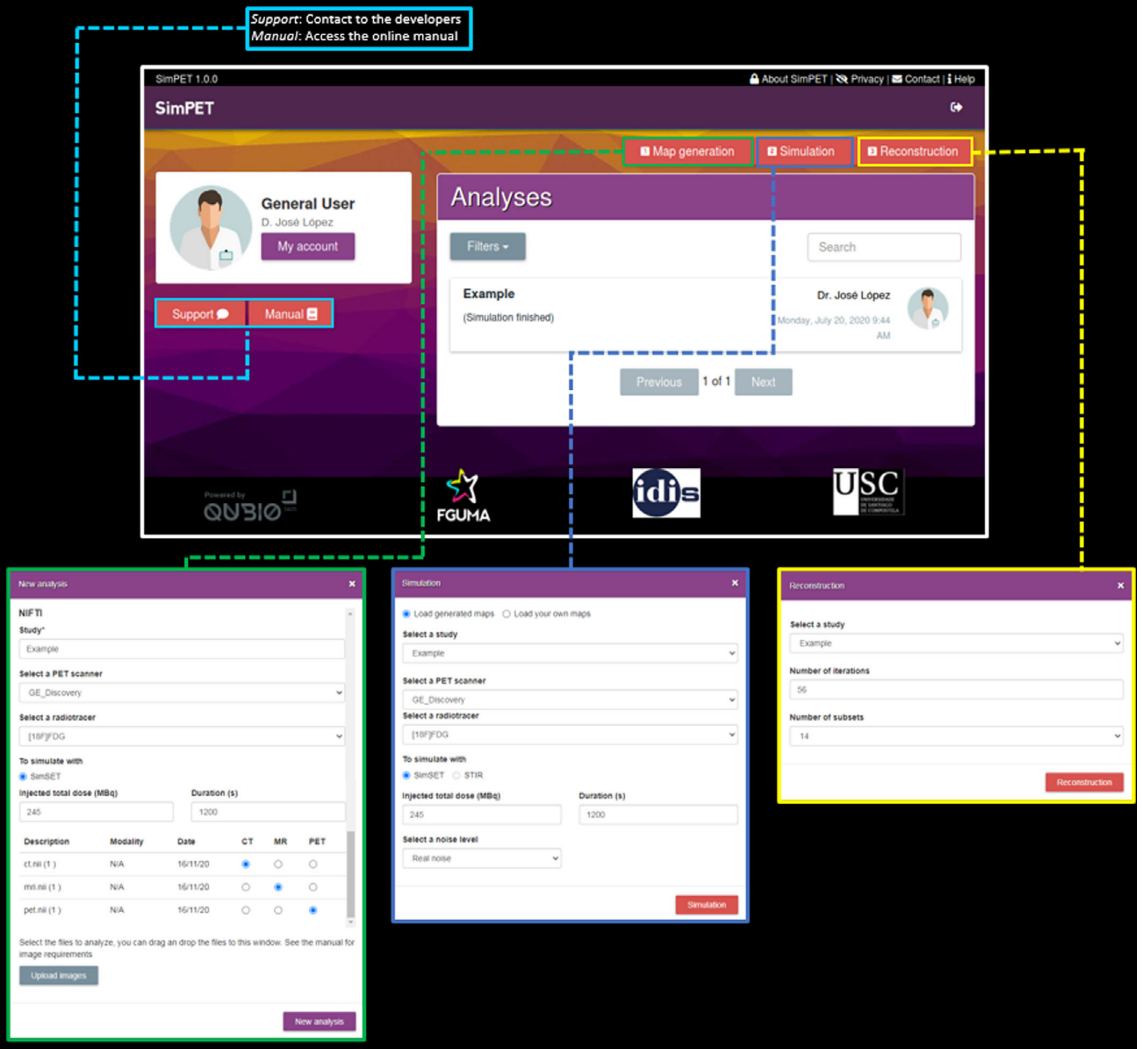
Web‐based Graphical User Interface of SimPET (www.sim‐pet.org). Surrounding boxes show the pop‐up menus for patient‐derived map generation (green), Monte Carlo simulation (dark blue), Tomographic reconstruction (yellow) and we highlight the different support options (light blue).

Figure 2 shows the GUI, that users can access freely through www.sim‐pet.org, after filling a registration form with their email and some additional information. The main menu is structured in three modules: Phantom generation (map generation), simulation, and reconstruction. After each process, intermediate files can be downloaded/uploaded, allowing the user to introduce extra steps in the process.^20^, ^21^ An interactive online image viewer is also included for evaluating the results without the need of downloading any file. The different boxes in Fig. 2 provide an overview of the different interaction panels in the platform. The user profile, an online manual and online support can also be found in the main page.

#### 2.A.4 Typical workflow

**Fig. 3:**
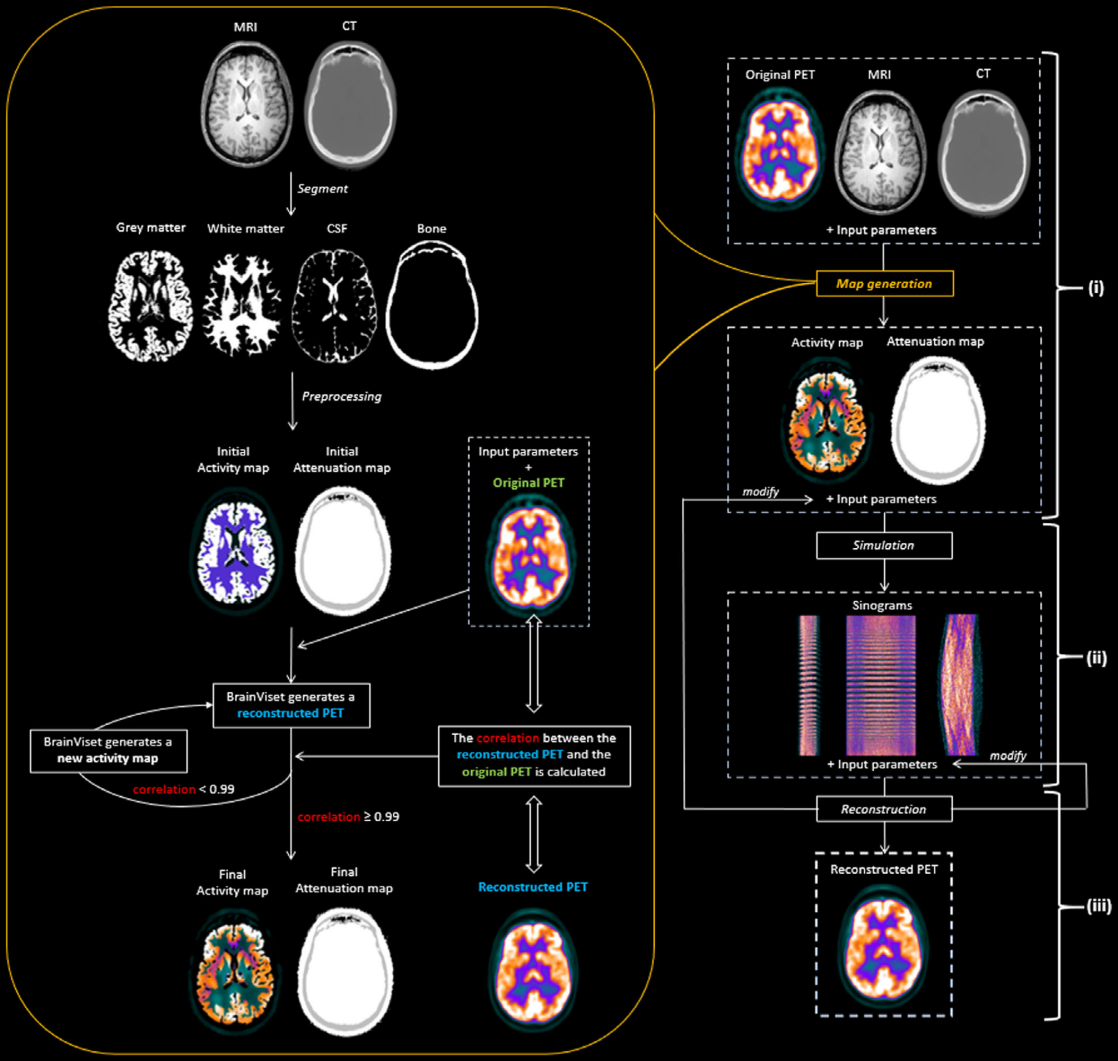
Typical workflow of SimPET. (i) First PET/CT and MRI images are considered as inputs for the activity and attenuation map generation. (ii) Next, the attenuation and activity maps are simulated for obtaining PET sinograms. (iii) Finally, the reconstructed PET images are generated by reconstructing these sinograms. The simulation and reconstruction steps can be repeated after changing the input parameters until the final image is satisfactory.

A typical workflow is illustrated in Fig. 3, including (a) loading PET, CT, and MR images of the same subject, as input parameters, and configuring a scanner model, a radiotracer, injected dose (MBq), and scan duration (s) for generating activity and attenuation maps using Brain‐VISET; (b) generating simulated sinograms from previously generated maps or from uploaded attenuation and activity maps using SimSET. For these, the user must set the desired injected dose (MBq), the scan duration (s), and the noise level, which can be allowed to easily generate noise‐free simulations independently of the selected parameters; and finally (c) reconstructing the above sinograms using STIR. Once the different images are generated, these can be viewed online or downloaded. For more details or an usage example, see the online manual at www.sim‐pet.org (Fig. 2, light blue box).

#### 2.A.5 Simulation and reconstruction

The MC simulation is performed using SimSET (v.2.9.2), which includes the simulation of all the physical processes for the energies of interest in nuclear medicine (below 1 MeV).^16^, ^38^ The generated data are then reconstructed by using STIR (v.3.1) (https://github.com/UCL/STIR). Currently, three commercial PET scanners are supported, namely the GE Discovery ST (GE Healthcare, Chicago, United States), The GE Advance NXi (GE Healthcare, Chicago, United States) and the Siemens Biograph mCT (Siemens Healthineers, Erlanger, Germany). Additional scanners will be progressively added in the future, as they get validated. The three scanner models are based on previously published works,^39^, ^40^, ^41^ and validated by us against experimental measurements taken with the GE Discovery ST present at CIMES (Centro de Investigaciones Médico‐Sanitarias, University of Málaga), the GE Advance NXi present at the University Hospital of Santiago de Compostela, and with published measurements for the Siemens Biograph mCT.^42^ The images are reconstructed using the ordered subsets expectation maximization (OSEM) algorithm as implemented in STIR, setting the reconstruction parameters to match those set in the scanner as closely as possible.

#### 2.A.6 Generation of realistic phantoms

Realistic activity and attenuation maps including nonuniform activities and physiological variability can be fully automatically extracted from patient images by using the Brain‐VISET iterative method, which has been previously presented in detail^20^, ^21^, ^29^ and can be seen schematically in the orange box of Fig. 3. In brief, PET/CT images are coregistered to the T1‐weighted MRIs using FLIRT^33^ (https://fsl.fmrib.ox.ac.uk/fsl). Bone tissue images are extracted from the CTs using a 600 Hounsfield unit threshold. The T1 MRI is segmented to gray matter, white matter, and cerebrospinal fluid using SPM12. An initial activity map is generated by filling the segmented tissues with uniform activities. An initial attenuation map is created joining the bone tissue and outskin images. The initial maps are then simulated using the MC model for the selected scanner. Postsimulation, the reconstructed image is compared with the original PET image, and the activity map is updated. This process is repeated iteratively until the correlation coefficient is ≥0.99.

### 2.B Validation

The validation was based on the simulation of realistic healthy patient’s FDG‐PET images databases for the included scanner models. To this end, FDG‐PET/CT and MRI images from 25 healthy subjects were scanned with the GE Discovery ST and used for the phantom generation. The generated activity and attenuation maps were then used as inputs for the simulation of synthetic data using the included GE Discovery ST, GE Advance NXi, and Siemens Biograph mCT scanner MC models. The validation was carried out by performing different comparisons between the simulated images and real healthy subject FDG‐PET images acquired on each of the scanners.

#### 2.B.1 Patient cohorts: PET and MRI acquisition protocols

FDG‐PET data acquisitions were performed as:

a. *Group 1:* 25 healthy subjects (mean age: 58 ± 5 yr; range: 48–67 yr) were acquired on a GE Discovery ST PET/CT installed at Centro de Investigaciones Médico‐Sanitarias (Málaga, Spain). Images were acquired for a bedtime of 1200 s after the intravenous injection of approximately 245 MBq (3.3 MBq/Kg) of ^18^F‐FDG. PET images were reconstructed using 3D OSEM with CT‐based attenuation correction, and scatter correction (Voxel size, 1.95 × 1.95 × 3.27 mm; Matrix size, 128 × 128 × 47). Furthermore, subjects also underwent MRI studies performed on a 3‐T MRI scanner (Philips Intera, Best, The Netherlands). High‐resolution T1 structural images of the whole brain were acquired with three‐dimensional (3D) magnetization prepared rapid acquisition gradient echo (3D‐MPRAGE) sequence.
b. *Group 2:* 25 healthy controls (mean age: 60 ± 4 yr; range: 54–65 yr) were acquired on a GE Advance NXi PET scanner present at the University Hospital of Santiago de Compostela (Santiago de Compostela, Spain). Images were acquired for 1200 s on 3D mode (no septa) after the injection of 370 MBq (4.7 MBq/Kg) of ^18^F‐FDG. PET images were reconstructed using 2D OSEM after attenuation (using a ^68^Ge source), scatter, and randoms precorrection and FORE rebinning (Voxel size, 2.05 × 2.05 × 4.3 mm; Matrix size, 128 × 128 × 35).
c. *Group 3:* 25 healthy controls (mean age: 53 ± 10 yr; range: 35–66 yr) were acquired on the Siemens Biograph mCT PET scanner at the Hospital Clinic (Barcelona, Spain), after injecting a dose of 185 MBq (2.5 Mbq/Kg) of ^18^F‐FDG, with a bedtime of 900 s. The images were reconstructed using time‐of‐flight (TOF) OSEM including resolution recovery (TrueX), attenuation, scatter, random, dead time, and decay corrections. (Voxel size, 1.02 × 1.02 × 1.50 mm; Matrix size, 400 × 400 × 148).

#### 2.B.2 Bland‐Altman comparison analysis

The acquired and simulated PET studies were spatially normalized onto the MNI space (voxel size of 2 × 2 × 2 mm) using a ^18^F‐FDG template and the 12‐parameter affine normalization (“Old Normalize”) provided by SPM12. The normalized PET studies were smoothed with a Gaussian kernel of 8 mm. For each scanner, the simulated images were compared with the real images by performing a Bland‐Altman‐like analysis, where for each voxel, we calculated voxel‐based differences ($\in_{\mathrm{voxel}}$) as:

$$\in_{\mathrm{voxel}}\;=2\;*\;\frac{\left(V_{\mathrm{real}}‐V_{\mathrm{simulated}}\right)}{\left(V_{\mathrm{real}}+\;V_{\mathrm{simulated}}\right)}$$

where $V_{\mathrm{real}}$ is the average value of the real images and $V_{\mathrm{simulated}}$ is the average value of the simulated images. The resulting values were histogrammed for presentation purposes. In order to considerer potential differences between the patient databases, the same process was performed between the real databases for comparison and included in the corresponding histograms when needed.

#### 2.B.3 Voxel‐wise comparison between simulated and acquired images

To complement the aforementioned comparison, the simulated images and real databases were compared by using SPM statistical analysis, which allows to assess regional/systematic differences between the simulated/real groups. As the activity maps were derived from *Group 1*, GE Discovery ST simulated images were compared using a paired *t* test configuration. For the rest of comparisons, we used a two‐sample *t* test. Two different contrasts were used to assess areas where the control group > simulated group and where the control group < simulated group. A statistic threshold of *P* < 0.01 and a cluster size k = 300 was applied. Family wise error (FWE) correction was applied to assess for multiple comparisons.

## 3 RESULTS

### 3.A Generation of digital phantoms

**Fig. 4:**
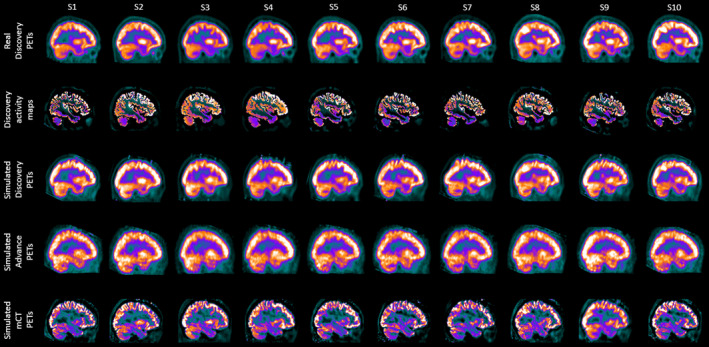
Sample of ten healthy control PET images acquired on GE Discovery ST (Group 1), the corresponding generated digital phantoms, and the corresponding simulated PET images obtained in the different commercial scanners using SimPET.

The CPU time for the generation of digital phantoms was about 3–4 h for GE Discovery ST, reaching convergence after four or five BrainVISET iterations. Figure 4 shows a subset of *Group 1* (row 1) and the corresponding generated digital phantoms (row 2).

### 3.B Simulation and reconstruction

The computation time for the simulation of the generated phantoms was about 50 min per subject, while the reconstruction time was 20 min for the GE Discovery ST and GE Advance NXi, and 40–60 min for the Siemens Biograph mCT scanner. This difference in reconstruction times was caused by the difference in times for the calculation of attenuation sinograms between the different scanners. Figure 4 (rows 3–5) shows the simulations of a subset of Group 1 in the three different scanners.

### 3.C Bland‐Altman comparisons

**Fig. 5:**
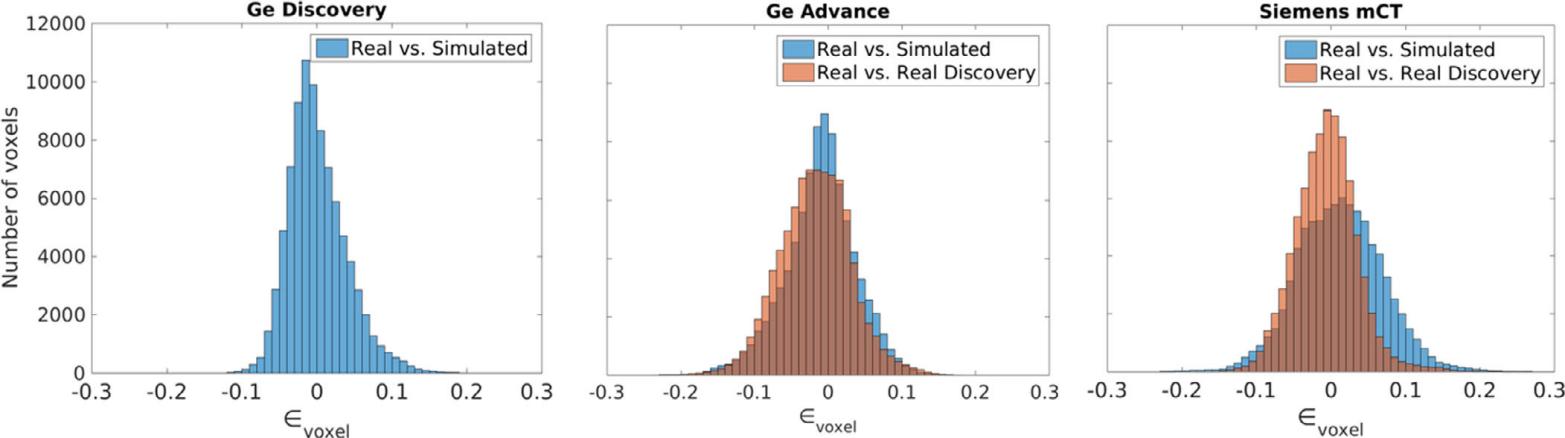
Bland‐Altman comparison between the real and simulated databases, for the GE Discovery ST (left), the GE Advance (center) and the Siemens mCT (right).

Figure 5 shows the results of the performed Bland‐Altman‐like analysis.

#### 3.C.1 GE discovery ST

In the left histogram, we can observe the differences between the real and the simulated images for the GE Discovery ST. 83.02% of voxels showed differences of <5%, while 98.09% were below 10%.

#### 3.C.2 GE advance NXi

For the GE Advance NXi (center), 71.98% of the voxels showed differences lower than 5%, while 95.09% were below 10%. These results were similar when comparing between real databases for the GE Discovery ST and the GE Advance NXi (68.42% voxels have differences lower than 5%, 94.55% are below 10%), pointing to the fact that most of the observed differences were due to differences between the databases.

#### 3.C.3 Siemens biograph mCT

Finally, for the Siemens Biograph mCT (right), 62.22% of the voxels showed differences of less than 5%, while 91.35% were below 10%. Differences between the real databases below 5% for 79.03% of the voxels, and below 10% for 97.60%, showing that the mCT provides the worst correspondence along the used scanner models.

### 3.D Voxel‐wise comparisons

**Fig. 6:**
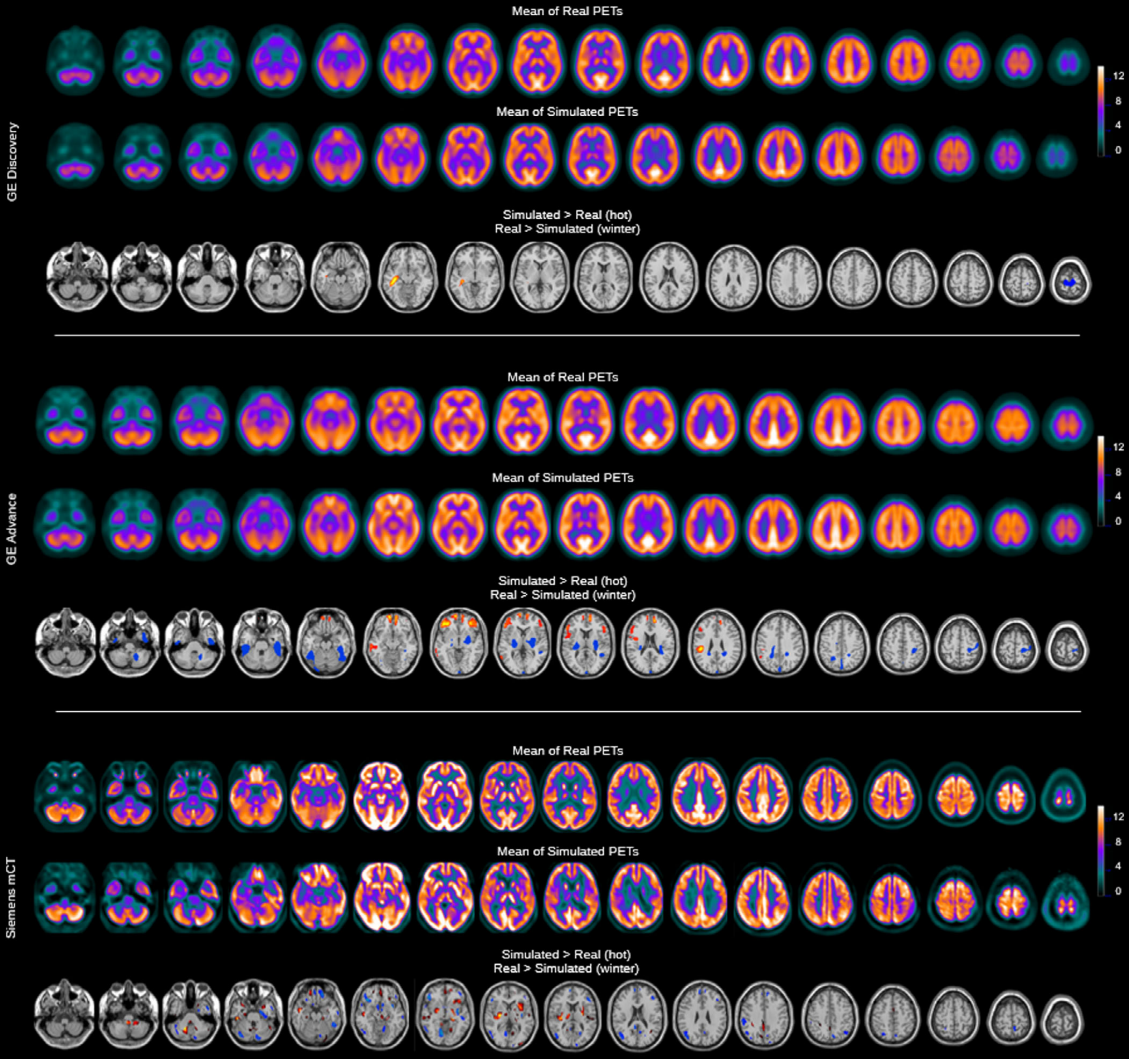
Voxel‐wise analysis results for the comparison between the simulated images and real FDG‐PET images for the three simulated scanners. A threshold of *P* < 0.01 corrected and k = 300 is applied.

Figure 6 shows the voxel‐wise statistical comparisons (mean and statistical differences) between acquired and simulated images for the different scanners.

#### 3.D.1 GE discovery ST

As it can be observed in Fig. 6 (rows 1–2), good visual agreement between the mean simulated and acquired images was achieved. Voxel‐wise analysis (Fig. 6, row 3) showed some regional differences, including regions of higher activity in the right temporal lobe of the simulated group and a bilateral region showing lower activity for the simulated group in the upper frontal lobe.

#### 3.D.2 GE advance NXi

As for the GE Discovery ST, a good visual agreement between simulated and real images is observed (Fig. 6, rows 4–5). The voxel‐wise analysis showed some areas of higher metabolism for the simulated group on the frontal lobe, while reduced activity was observed in the temporal lobe and the internal structures (Fig. 6, row 6).

#### 3.D.3 Siemens biograph mCT

For the Siemens Biograph mCT we observed the greater visual differences between the simulations and the acquisitions (Fig. 6, rows 7–8). Artifacts can be observed in the simulated images, such as slight asymmetries on the occipital and temporal lobes that are not observed for the other scanners. The voxel‐wise analysis showed higher simulated activity in the cerebellum, the internal structures and some small cortical areas in the right frontal lobe and the left occipital lobe. In contrast, we observed reduced simulated metabolism in several small clusters distributed along the cortex.

## 4 DISCUSSION

The evaluation and standardization of quantification methods in brain PET require the analysis of large pools of PET images, ideally against a well‐known ground truth. While physical phantoms provide a way forward, acquiring many phantom images can be tedious and time consuming. In addition, physical phantoms are often limited to simple geometric shapes, leading to unrealistic images. Therefore, simulations can be of value in this case. As a rule, however, great technical expertise and high computing power are required. In recent works, easy‐to‐use platforms for analytical simulation^43^ were presented. In this work, we expand the range of available options by offering a free, intuitive, and efficient tool for Monte Carlo (MC) simulation of realistic PET images, with a focus on brain imaging. Our platform, SimPET, simplifies the process of obtaining realistic synthetic brain PET images by combining tools for extraction of digital phantoms from patient data and well‐validated scanner models.

To demonstrate the capabilities of the platform, we generated synthetic databases of healthy patients for the three scanner models included in the library. For this, we generated realistic activity and attenuation maps derived from 25 healthy patients acquired on the GE Discovery ST, that were then simulated using different scanner models. Despite the images, generated by the platform, being visually close to the real images for all scanners (see Fig. 4), statistical analysis revealed significant differences, especially for the GE Advance and the Siemens mCT. In this regard, quantitative analysis was performed by comparing with the real images acquired on each scanner by a) Bland‐Altman analysis and b) SPM statistical analysis (see Figs. 5 and 6). In both cases, the GE Discovery ST showed the smallest differences between the acquired and simulated images (83.02% of voxels have differences of <5%). This is not surprising, as this was the most favorable comparison, where the simulated images were compared with the FDG‐PET images from which the digital phantoms were derived. Results of the other scanners (GE Advance NXi and Siemens Biograph mCT) showed bigger differences. While these differences could be attributed to physiological differences between the control groups (see Fig. 5), more work will be needed to validate this hypothesis, or to improve the MC models for these tomographs in order to diminish these differences. Overall, good agreement between simulated and acquired data opens the door for using the platform for applications such as augmenting data for training artificial intelligence algorithms and others. In addition to the images generated from healthy patients, Appendix A includes complementary experiments. Despite no quantitative analysis being presented, these experiments may serve as examples to highlight the potential applications of the platform for performing simulations of different tracers and to present the users with appropriate workflows that might be used for validating/harmonizing quantification protocols.

Despite the overall good performance, the current version of the platform presents some limitations, particularly relevant to the Siemens Biograph mCT model. First, the simulation is based on a simplified detector model (SimSET simplePET). While this allows for short simulation times and might be sufficient for old scanners, modern scanners would benefit from more detailed models. Second, our reconstruction does not include features such as TOF and resolution recovery. These features are currently under development and will be available with a future release.^44^ In addition to these features, novel platform tools which will allow the users to easily introduce new scanner models will be added. Our goal is to build an open and diverse scientific community of users and developers.

## 5 CONCLUSIONS

SimPET is an open, efficient, and user‐friendly online platform for the generation of synthetic brain FDG‐PET datasets. Comparisons between generated and acquired data showed reasonable agreement for the simulations, especially for the GE Discovery ST, demonstrating that SimSET can be used for generating realistic simulated data. Further work needs to be performed to validate map generation and simulation on other scanner models.

## CONFLICT OF INTEREST

JSR and PA are advisors for Qubiotech Health Intelligence SL. The authors have no other relevant conflict of interest to disclose.

## Figures and Tables

**Fig. A1:**
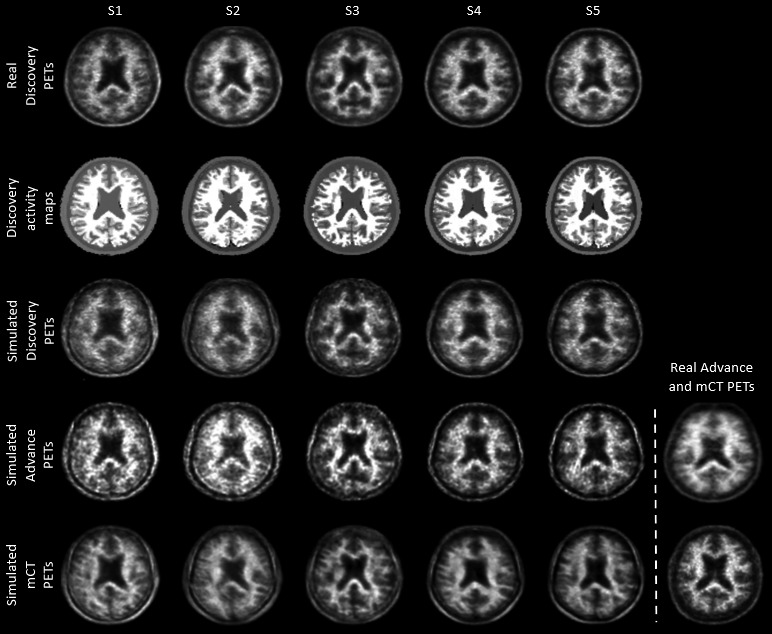
Sample of five amyloid‐negative patient PET images acquired on GE Discovery ST, the corresponding generated activity maps, and the simulated PET images obtained in the different commercial scanners, GE Advance and Siemens mCT images are included for comparison.

**Fig. A2:**
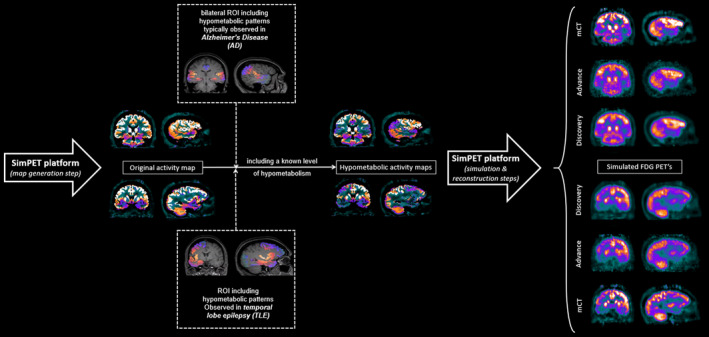
Pipeline used to simulate pathological PET images of AD and TLE cases using activity maps generated on SimPET and including a known level of hypometabolism.

## Data Availability

The data generated and/or analyzed during the current study are available from the corresponding author on reasonable request. The activity maps used in the present study are available through the SimPET platform (www.sim‐pet.org). The code used for the Monte Carlo simulation is available on Github at https://github.com/txusser/brainviset_simset.

## Appendix A

### AMYLOID PET SIMULATION

In addition to ^18^F‐FDG, the platform also allows users to simulate amyloid PET images with ^18^F‐Florbetapir as a tracer. For demonstrating this capability, we include a simple example case. Five amyloid‐negative patients, acquired on the GE Discovery ST, were uploaded to the platform to obtain amyloid PET realistic activity maps. These maps were then simulated on the three implemented scanners and the results are showcased for demonstration purposes, showing the differences between scanners in amyloid PET assessments.

Figure A1 shows the results of the performed amyloid PET simulations. Five amyloid‐negative PET patients and the obtained activity maps are shown in rows 1 and 2, followed by the simulated PETs obtained with the platform for the GE Discovery ST, GE Advance NXi and Siemens Biograph mCT scanner models (rows 3–5). Real amyloid‐negative PET examples for the GE Advance NXi and Siemens Biograph mCT scanners are included for comparison. For additional information on how simulated amyloid images can be used for validating quantification methods, readers can check our previous work.^21^

### SIMULATION OF PATHOLOGICAL MAPS

In order to validate quantitative methods in different pathologies, users can download the generated activity maps, and modify them to introduce known hypometabolism patterns, which can be used as ground truth for the quantification after the simulation and reconstruction. As an example, one of the generated ^18^F‐FDG activity maps was downloaded, and two different hypometabolism were added manually in predefined ROIS reflecting patterns typically observed in Alzheimer’s disease (AD) and temporal lobe epilepsy (TLE). The simulations of these patterns in the three scanners are showcased in Figure A2, allowing for the different representation of the exact same metabolism in the different scanners to be easily observed.

After simulating the generated patterns using the platform, measurements on the different scanners can be compared, allowing the users to validate quantification and harmonization protocols. A detailed example on how to exploit this procedure can be found in our previous work.^20^
